# Ethnic-Racial Socialization, Teacher Discrimination, and Black Youth’s School Engagement and Achievement

**DOI:** 10.1007/s11121-023-01551-z

**Published:** 2023-06-07

**Authors:** Sharon F. Lambert, Farzana T. Saleem, Chang Liu, Theda Rose

**Affiliations:** 1 Department of Psychological and Brain Sciences, The George Washington University, 2013 H Street NW, Washington, DC, 20006 USA; 2 Graduate School of Education, Stanford University, Stanford, USA; 3 Department of Psychology, Washington State University, Pullman, USA; 4 School of Social Work, University of Maryland Baltimore, Baltimore, USA

**Keywords:** Ethnic-racial socialization, Teacher discrimination, Black adolescents, School engagement, Academic achievement

## Abstract

Ethnic-racial socialization is one strategy Black parents use to support their children’s school engagement and academic achievement given the occurrence and toxic effects of discrimination. Egalitarianism and preparation for bias socialization messages have yielded mixed evidence of promotive and protective effects for Black youth's school outcomes, and effects may vary according to ethnicity. Thus, this research examined associations between ethnic-racial socialization messages and school engagement and achievement, and whether these messages protected against teacher discrimination effects on academic achievement transmitted through school engagement, among a nationally representative sample of Black adolescents who participated in the National Survey of American Life Adolescent supplement study. Ethnic-racial socialization message content and the frequency of communication about race demonstrated different associations with engagement (i.e., school bonding, aspiration-expectation discrepancy, and disciplinary actions) and achievement (i.e., grades) for African American and Caribbean Black youth. However, the benefits were not sufficient to combat the adverse effects of teacher discrimination on school engagement and, in turn, achievement. These findings highlight the utility of integrating ethnic-racial socialization into prevention programs to support Black youth’s school experiences; demonstrate the importance of attention to heterogeneity within Black youth; and underscore the critical need for prevention programs to address teacher discrimination.

**Supplementary Information:**

The online version contains supplementary material available at 10.1007/s11121-023-01551-z.

Despite some reductions in racial gaps in educational achievement and attainment, educational success remains a prevailing challenge for Black youth; they are disproportionately represented among youth who experience lower achievement, greater suspension and grade retention, and lower educational expectations by teachers, and attend schools with depleted financial resources (Braun et al., 2010; Clotfelter et al., 2009; Musu-Gillette et al., 2017). Compounding these concerns, Black youth encounter racism in the school context that has been linked with a multitude of negative school experiences and poor educational outcomes (e.g., McFarland et al., 2018). Given evidence that poor educational experiences forecast worse emotional and behavioral health, lower occupational attainment, lower economic well-being, and increased morbidity and mortality (e.g., Musu-Gillette et al., 2017; U.S. Bureau of Labor Statistics, 2018), it is clear that Black youth’s disparate experiences in the school context fuel racial health disparities beginning in adolescence and persisting over the life course.

Because teacher discrimination is prevalent in Black youth’s school experience and a daily reality (e.g., English et al., 2020),  youth often encounter multiple types of ethnic-racial socialization (ERS) messages. Among these are messages emphasizing equal rights and opportunities across race and ethnicity (egalitarianism) and messages that increase their awareness of discrimination and provide strategies for coping with it (preparation for bias) (Hughes et al., 2006). Understanding how ERS messages affect Black youth’s educational experiences is needed for the design and refinement of prevention interventions aimed to promote optimal developmental outcomes for Black youth. Thus, the purpose of this study is to examine how egalitarianism and preparation for bias messages are associated with Black adolescents’ school engagement and achievement, and whether these ERS messages attenuate associations between teacher discrimination and educational experiences of Black youth. We explore these associations among African American and Caribbean Black youth.

This research is guided by Garcia Coll and colleagues’ integrative model of minority child development (1996) and Bronfenbrenner’s ecological model (Bronfenbrenner & Morris, 2006), each of which highlight race, ethnicity, and discrimination as factors central in understanding Black youth’s developmental outcomes. These theories, along with the Cultural Asset Framework (Gaylord-Harden et al., 2012), highlight ethnic-racial socialization messages as important for promoting youth’s optimal outcomes and offering protection against race-related stress. According to the integrative model, social stratification has implications for Black youth’s experience of race-related stressors such as discrimination, the socialization messages they receive, and their developmental outcomes. Similarly, the Cultural Asset Framework and multiple theorists (e.g., Hughes et al., 2006), emphasize that attention to culturally specific assets that support youth is important for identifying strength-based and resilience-oriented factors to support development and safeguard Black youth against race-related stress.

## Black Youth’s Educational Experiences

School engagement is central in understanding Black youth’s school outcomes. Scholars agree that school engagement is multidimensional, including three dimensions: affective or emotional engagement, cognitive engagement, and behavioral engagement (e.g., Christenson et al., 2012). Affective engagement has been conceptualized as one’s sense of connection and belonging at school and is consistently linked with better academic outcomes (Lei et al., 2018). Cognitive engagement encompasses students’ beliefs about themselves, including their academic aspirations and expectations. While both are relevant for academic outcomes, for youth from marginalized backgrounds, aspirations and expectations may not align as these youth experience and observe inequity in schooling experiences, educational attainment, and occupational status based on race. Adolescents whose academic aspirations exceed their expectations have poorer academic adjustment (Boxer et al., 2011) than youth whose aspirations and expectations are congruent, suggesting that aspiration-expectation discrepancies are an important index of cognitive engagement for Black youth. Behavioral engagement includes participation in school and learning activities, as well as students’ conduct. Not surprisingly, disciplinary actions that remove students from the learning environment forecast poorer school performance and achievement. Black youth experience disproportionate disciplinary actions including office referrals, suspension, expulsion, and referral to juvenile justice when compared to youth of other racial backgrounds (e.g., Riddle & Sinclair, 2019). These experiences interrupt classroom time, present multiple challenges to Black youth’s academic outcomes (Gregory et al., 2010), and play a role in perpetuating the damaging trajectory from school to prison.

Teacher discrimination has been associated with less school bonding (e.g., Kyere et al., 2020), more disciplinary actions (Butler-Barnes et al., 2020), and poorer academic achievement (Assari & Caldwell, 2018). Also, reduced school engagement has been identified as a mechanism linking teacher discrimination and lowered achievement (Bryan et al., 2018). Parallel findings have been found for school racial climate and teacher support. For example, (Griffin et al., 2017) found that cognitive and behavioral engagement mediated between school racial climate and African American high school students’ academic achievement.

## Ethnic-Racial Socialization and Black Youth’s Educational Experiences

Ethnic-racial socialization (ERS), the verbal and nonverbal messages and behaviors communicated about race, ethnicity, and discrimination, can influence how Black youth process and respond to race-based experiences. ERS can provide youth with tools on how to understand and respond to racialized experiences within and beyond the school context. Two common, yet somewhat contrasting, messages are those that alert youth about racial discrimination and how to cope with it (i.e., preparation for bias) and those that minimize racial differences and instead focus on individual characteristics (i.e., egalitarianism) (Hughes et al., 2006).

Research examining ERS messages and Black youth’s educational experience and academic performance has yielded mixed results varying according to the message content and the outcome examined (Wang et al., 2020). For example, greater preparation for bias has been related to greater school bonding and higher school self-esteem for African American youth (Dotterer et al., 2009). Likewise, moderate levels of racial barrier socialization, conceptualized as awareness of racism and discrimination, were associated with better academic self-esteem among a sample of African American adolescents (Cooper & Smalls, 2010). On the other hand, preparation for bias has been associated with lower academic performance (e.g., Smith et al., 2003) and not associated with grades (Murry et al., 2009; Neblett et al., 2006) in other research. Similarly, disparate findings have emerged for egalitarianism messages. More egalitarianism messages have been associated with greater academic curiosity and academic persistence in some research (Neblett et al., 2006), but other research has shown that these socialization messages are associated with lower school self-esteem for Black youth (Constantine & Blackmon, 2002). Others have found that adolescents whose parents’ socialization messages included a high amount of egalitarianism messages reported more positive academic self-beliefs than adolescents whose parents’ socialization messages were mainly characterized by preparation for bias (Metzger et al., 2020).

Evidence of ERS protecting against school discrimination has been mixed. (Banerjee et al., 2018) found that preparation for bias protected African American adolescents against the effects of school-based discrimination by peers on academic persistence, academic self-concept, and perceived academic self-efficacy. On the other hand, others have found that ERS messages support multiple academic outcomes such as academic curiosity, academic persistence, school bonding, and grades for African American youth, but do not protect against the effects of discrimination on these outcomes (Dotterer et al., 2009; Neblett et al., 2006).

Whether egalitarianism and preparation for bias messages support Black youth’s academic adjustment may vary by ethnicity given differences in distinct social and historical experiences and cultural values (e.g., Levine et al., 2015). Prior evidence suggests that there may be differences in how African American and Caribbean Black families perceive and prepare for structural barriers. For example, compared to African American families, Caribbean Black families are less likely to highlight race as a barrier to social mobility and more likely to minimize racism in their socialization efforts (e.g., Hunter, 2008). These differences suggest variation in the frequency of preparation for bias and egalitarianism messages African American and Caribbean Black youth encounter; it also is possible that these messages are associated differently with youth outcomes given their different salience in these ethnic groups.

## Current Study

The overall aim of this research is to examine the promotive and protective effects of ERS messages for Black youth’s school engagement (i.e., school bonding, aspiration-expectation discrepancy, and disciplinary actions) and academic achievement (i.e., grades). To achieve this aim, there are three sub-aims: (1) to examine the association between ERS messages and engagement and grades; (2) to examine a hypothesized mediational mechanism whereby the negative impact of teacher discrimination on academics is mediated by student engagement; (3) to examine whether the pathway between discrimination, engagement, and educational outcomes is moderated by ERS messages.

We focus on preparation for bias and egalitarianism messages as these messages have shown less consistent results than cultural socialization messages in prior research, and because egalitarianism messages have received scant empirical attention despite their frequent use in educational settings (Wang et al., 2020). In addition, we consider whether the frequency of discussions about race, irrespective of message content, benefits Black youth’s educational experience and academic achievement. For sub-aim 1, we hypothesized that the promotive effects of ERS messages on school engagement and grades would vary by message type. We expected that egalitarianism would be associated with more school bonding and preparation for bias with less school bonding. We expected that egalitarianism would be associated with smaller discrepancies between academic aspirations and expectations, but preparation for bias with larger discrepancies. We did not have a priori hypotheses about how egalitarianism and preparation for bias would be associated with disciplinary actions because ERS messages likely influence how youth respond to, but not influence whether they experience, disciplinary actions. We hypothesized that both egalitarianism and preparation for bias messages would be associated with a higher grade point average. In contrast to predictions about ERS message content, we expected that more frequent discussions about race generally would show similar associations as preparation for bias as discussions about race likely raise awareness and vigilance to manage racial stressors youth may encounter.

We examined the protective effects of ERS messages in the context of an indirect pathway linking teacher discrimination and grades through engagement (i.e., school bonding, aspiration-expectation discrepancy, and disciplinary actions). For sub-aim 2, we expected that like lower school bonding (e.g., Bryan et al., 2018), larger aspiration-expectation discrepancies and more disciplinary actions would link teacher discrimination and lower grade point average. For sub-aim 3, we hypothesized that preparation for bias messages and a greater frequency of discussions about race would interrupt the pathway from teacher discrimination to school engagement and grades, because youth would be more aware and better prepared to manage discrimination in the school context. We did not expect that egalitarianism messages would be similarly protective.

Throughout, we considered ethnic group heterogeneity in our sample. Native-born and immigrant-born Black youth may receive different treatment within school settings and may be socialized differently about how to manage such treatment. In addition, the protective effects of ERS messages may vary by ethnicity. For example, (Saleem et al., 2022) found that for Caribbean Black youth, but not African American youth, the association between discrimination and perceived stress was stronger in the context of more preparation for bias messages. Thus, we hypothesized that the protective effects of preparation for bias messages would be evident for African American youth but not Caribbean Black youth.

## Method

### Design and Sample

This study utilizes extant data from the National Survey of American Life – Adolescent Supplement (NSAL-A, 2001–2004). The NSAL, a nationally representative household survey, was approved by the Institutional Review Board (IRB) at the University of Michigan (Jackson et al., 2004). Using a stratified and clustered sample design, African American (AA) and Caribbean Black (CB) households included in the NSAL were screened; eligible adolescents residing in the household were randomly selected for participation. The distinction between African American and Caribbean Black was based on the ethnicity of the adult household from which the adolescent participant was drawn. Specifically, AA participants were those individuals who identified as Black with no ancestral ties to the Caribbean. CB participants were those who identified as Black with ties to the Caribbean through indicating (a) they were of Caribbean or West Indian descent; (b) their parents or grandparents were born in a Caribbean country; or (c) they were from a Caribbean country from a list provided to them by the interviewer (Heeringa et al., 2004; Jackson et al., 2004; Seaton et al., 2008). Only about 20% of the CB youth had parents born in the USA.

If multiple adolescents in the household were eligible, up to two were selected for the study, with consideration for gender. The NSAL-A was weighted to adjust for variation in probabilities of selection within households, and nonresponse rates for adolescents and households. The weighted data were post-stratified to approximate the national population distributions for gender, age, and ethnicity. Acquisition of informed consent from the adolescent’s legal guardian and assent from the adolescent preceded all interviews. Most interviews were conducted in the adolescent’s homes with 18% conducted by telephone. Respondents were compensated $50 for their participation in the study; the overall response rate was 80.6% (80.4% for AAs and 83.5% for CBs). The final sample included 1170 AA (*n* = 810) and CB (*n* = 360) youths, 13 to 17 years old (Seaton et al., 2010).

### Measures

#### Ethnic-Racial Socialization

The Content Scale of the Comprehensive Race Socialization Inventory (Lesane-Brown et al., 2005) was used to assess youth’s exposure to preparation for bias messages and egalitarianism messages. Two items (e.g., “Ever told hard work achieved anything”; “Ever told race does not matter”) reflected egalitarianism. Three items (e.g., “Ever told whites think they are above blacks”; Ever told you will experience racism”; “Ever told need to act white to get ahead”) reflected preparation for bias. A sum was calculated for each message type; scores ranged from 0 to 2 for egalitarianism, and from 0 to 3 for preparation for bias. Given the short scales, inter-item correlations were calculated as an indicator of reliability (Eisinga et al., 2013). Inter-item correlations ranged from *r* = 0.26 to *r* = 0.28 for preparation for bias; the inter-item correlation was *r* = 0.30 for promotion of mistrust and *r* = 0.33 for egalitarianism. Reliability for these subscales was similar to that of other short ERS scales (e.g., French et al., 2013).

#### Frequency of Race Communication

Participants reported how often they received messages about what it means to be Black and messages to help them deal with people outside of their race from parents, other relatives, friends, and other adults (e.g., “How often do your parents or the people who raised you talk with you about race or racism?”). Items were rated on a 5-point scale ranging from 1 (“Very often”) to 5 (“Never”). Items were reverse-scored and averaged such that higher scores indicate a higher frequency of discussions about race. The internal consistency was 0.82 and 0.75 for African American and Caribbean Black adolescents, respectively.

#### School Bonding

A 9-item scale derived from the National Comorbidity Survey: Adolescent Supplement (Kessler, 2011) was used to assess school bonding. Items assessing affective connection and attitude toward school (e.g., “Most of my teachers treat/ed me fairly”) were rated on a 4-point scale ranging from 1 (very true) to 4 (not at all true). Positively worded items were reverse-scored and averaged such that higher scores represent greater bonding to school. The internal consistency was 0.71 for African American and 0.70 Caribbean Black adolescents.

#### Discrepancy Between Aspirations and Expectations

Participants reported “how far they would like to go in school” (aspirations) and “how far they think they will get in school (expectations), with responses ranging from “not graduate high school” to “graduate/professional degree.” Aspiration-expectation (A-E) discrepancy scores were computed by subtracting expectation scores from aspiration scores. If the aspiration score was greater than the expectation score, the A-E discrepancy was coded as 1; if the aspiration score was equal to or lower than the expectation score, the A-E discrepancy was coded as 0. Similar methods for computing a dichotomous discrepancy score from the difference between aspirations and expectations have been used in prior research (e.g., Boxer et al., 2011).

#### Disciplinary Actions

Three items were used to measure school disciplinary actions: “Were you ever suspended from school for a day or longer?” “Were you ever expelled from school?” “Have you ever spent time in a reform school, detention center, jail, or prison?” A school disciplinary action score was computed by summing these items, yielding scores ranging from 0 to 3.

#### GPA

Grades were measured using participants’ self-report of the kinds of grades they usually receive (i.e., mostly As, mostly Bs, mostly Cs, mostly Ds, or mostly failing grades). The letter grades were converted into a 5-point scale (A = 5, B = 4, C = 3, D = 2, Failing = 1).

#### Perceived Teacher Discrimination

Three items were used to assess perceived teacher discrimination “Your teachers treat you with less respect than other students,” “Your teachers act as if they think you are not smart,” and “Your teachers act as if they are afraid of you.” Items were scored on a 6-point scale ranging from 1 “almost everyday” to 6 “never.” Items were reverse-scored and a mean score calculated such that higher scores indicate more perceived discrimination from teachers. The internal consistency was 0.73 and 0.70 for African American and Caribbean Black adolescents, respectively.

#### Demographic Variables

Adolescent age, adolescent sex, family income, and parental education were included in the analyses to account for possible confounds. Adolescents reported their age and sex. Family income and parent education data were merged from the NSAL adult data. Family income was measured on a 6-point response scale ranging from 0 ($0) to 6 (over $100,000). Parent education was a continuous variable assessing adult respondent (parent) years of school. Scores ranged from 0 to 17.

## Analytic Strategy

### Attrition Analysis

Of the 1170 linked sets of participants, the proportion of missingness is listed below: frequency of communication about race: 0.68%; preparation for bias: 10.09%; egalitarianism: 9.32%; teacher discrimination: 0%; school bonding: 0%; aspirations: 0.34%; discrepancy: 0.60%; disciplinary actions: 0%; and grades: 0.68%. Full information maximum likelihood (FIML) estimation was used to account for missing data.

### Data Analysis

All analyses were conducted separately for African American and Caribbean Black youth. Given the stratified and clustered sampling design, we used weights to adjust for nonindependence and nonresponse rates within households, across households, and among individuals. We used M*plus* to correct standard errors given the complex data.

Analyses proceeded in three steps. First, descriptive statistics (means, standard deviations, and bivariate correlations) of primary study variables were calculated. The promotive effects of ERS messages were assessed based on the bivariate correlations between ERS messages and school engagement and academic achievement outcomes. Next, we examined whether ERS protected in the indirect teacher discrimination → engagement → grades pathway. Thus, the second step was to examine whether adolescents’ school engagement (i.e., school bonding, A-E discrepancy, and disciplinary actions) mediated the association between teacher discrimination and adolescents’ grades after controlling for adolescent age, adolescent sex, family income, and parental education. The third step was testing moderation by examining the teacher discrimination → engagement → grades pathway under high- vs. low-ERS frequency and ERS message content (i.e., preparation for bias, egalitarianism) after controlling for covariates. Steps 2 and 3 were examined using structural equation modeling with M*plus* 8 (Muthén & Muthén, 2017). For step 2, we tested for indirect effects using bootstrapping with 10,000 iterations. Of note, when bootstrap was used, model fit information, including the chi-square test of model fit, CFI, TLI, and RMSEA, were not available in M*plus*.

## Results

### Promotive Effects of ERS Messages

Partial correlations among the primary study variables, by ethnicity and controlling for covariates (age, sex, family income, and parental education), are presented in Table 2 (see Supplementary Materials). For AA youth, preparation for bias was correlated negatively with school bonding (*r* =  − .11, *p* < .01) and positively with grades (*r* = .08, *p* < .05). Egalitarianism was correlated negatively with disciplinary actions (*r* =  − .11, *p* < .05) and positively with grades (*r* = .12, *p* < .01). The frequency of race communication was associated positively with school bonding (*r* = .08, *p* < .01). For CB youth, preparation for bias was correlated negatively with school bonding (*r* =  − .20, *p* < .01), positively with discrepancy (*r* = .12, *p* < .05), and positively with disciplinary actions (*r* = .19, *p* < .01). The frequency of race communication was correlated positively with school bonding (*r* = .14, *p* < .01), negatively with discrepancy (*r* =  − .14, *p* < .05), and positively with grades (*r* = .11, *p* < .05). Egalitarianism was not correlated with school engagement or grades for CB youth.

### Protective Effects of ERS Messages

Our test of the protective effects of ERS in the context of the teacher discrimination → engagement → grades pathway included three steps. First, we examined the association between teacher discrimination and grades after controlling for the covariates (i.e., adolescent age, adolescent sex, family income, and parental education). Second, we examined whether adolescents’ school engagement (i.e., school bonding, A-E discrepancy, and disciplinary actions) mediated the association between teacher discrimination and adolescents’ grades. Third, we conducted multigroup analyses to examine these pathways for (1) high- vs. low-egalitarianism groups, (2) high- vs. low-preparation for bias groups, and (3) high- vs. low-frequency of race communication; these multigroup analyses were to inform whether pathways differed for high vs low ERS, our strategy for investigating moderation.

#### Association Between Teacher Discrimination and Grades

For AA and CB youth, teacher discrimination was negatively associated with adolescents’ grades (AA: *β* =  − .07, *p* = .03; CB: *β* =  − .17, *p* < .01), such that higher teacher discrimination was associated with lower grades.

#### Teacher Discrimination → Engagement → Grades Pathway

For these analyses, school bonding, A-E discrepancy, and disciplinary actions were examined in separate mediation models.^1^ The results are presented in Fig. 1 (see Supplementary Materials).

#### School Bonding

For AA and CB youth, higher teacher discrimination was associated with lower school bonding (AA: *β* =  − .24, 95% CI [− .31, − .17]; CB: *β* =  − .44, 95% CI [− .58, − .38]), and higher school bonding was associated with higher grades (AA: *β* = .23, 95% CI [.14, .32]; CB: *β* = .45, 95% CI [.37, .55]). The indirect effect was significant for AA and CB youth, such that higher teacher discrimination was associated with lower grades through school bonding (AA: *β* =  − .06, 95% CI [− .08, − .03]; CB: *β* =  − .20, 95% CI [− .30, − .16]).

#### A-E Discrepancy

For AA and CB youth, higher teacher discrimination was associated with a higher A-E discrepancy (AA: *β* = .12, 95% CI [.02, .21]; CB: *β* = .13, 95% CI [.03, .27]). For AA and CB youth, higher A-E discrepancy was associated with lower grades (AA: *β* =  − .14, 95% CI [− .24, − .04]; CB: *β* =  − .16, 95% CI [− .32, − .02]). The indirect effect was significant for both AA and CB youth, such that higher teacher discrimination was associated with lower grades through its effect on A-E discrepancy (AA: *β* =  − .02, 95% CI [− .04, − .001]; CB: *β* =  − .02, 95% CI [− .04, − .003]).

#### Disciplinary Actions

For AA and CB youth, higher teacher discrimination was associated with more disciplinary actions (AA: *β* = .15, 95% CI [.03, .25]; CB: *β* = .31, 95% CI [.19, .42]). More disciplinary actions were associated with lower grades for AA youth ( *β* =  − .20, 95% CI [− .29, − .11] but not for CB youth (*β* =  − .15, 95% CI [− .29, .01]). The indirect effect was significant for AA youth; higher teacher discrimination was associated with lower grades through its effect on disciplinary actions ( *β* =  − .03, 95% CI [− .06, − .01]).

#### ERS Moderation of Teacher Discrimination → Engagement → Grades Pathway

Multigroup analyses were conducted to examine the associations between teacher discrimination and adolescents’ grades via adolescents’ school engagement under (1) high- vs. low-egalitarianism groups, (2) high- vs. low-preparation for bias groups, and (3) high- vs. low-frequency of race communication, respectively. Results for multigroup analyses are presented in Tables 3–5 (see Supplementary Materials).

#### School Bonding

For youth with high egalitarianism, for both AA and CB youth, higher teacher discrimination was associated with lower school bonding (AA: *β* =  − .19, 95% CI [− .28, − .09]; CB: *β* =  − .45, 95% CI [− .57, − .34]), and lower school bonding was associated with lower grades (AA: *β* = .24, 95% CI [.14, .33]; CB: *β* = .55, 95% CI [.43–.69]). In contrast, for youth with low egalitarianism, for both AA and CB youth, higher teacher discrimination was associated with lower school bonding (AA: *β* =  − .38, 95% CI [−.51, − .26]; CB: *β* =  − .45, 95% CI [− .59, − .21]), whereas lower school bonding was not associated with lower grades for CB youth or AA youth.

For youth with both high and low preparation for bias, for both AA and CB youth, higher teacher discrimination was associated with lower school bonding (high preparation for bias group: AA: *β* =  − .23, 95% CI [− .26, − .10]; CB: *β* =  − . 44, 95% CI [− .53, − .27]; low preparation for bias group: AA: *β* =  − .22, 95% CI [− .38, − .05]; CB: *β* =  − .44, 95% CI [− .63, − .11]), and lower school bonding was associated with lower grades (high preparation for bias group: AA: *β* = .23, 95% CI [.13, .33]; CB: *β* = .46, 95% CI [.38, 53]; low preparation for bias group: AA: *β* = .25, 95% CI [.06, .39]; CB: *β* = .61, 95% CI [.12, .82]). Similarly, for youth with both high and low frequency of race communication, for both AA and CB youth, higher teacher discrimination was associated with lower school bonding (high frequency of race communication: AA: *β* =  − .27, 95% CI [− .32, − .20]; CB: *β* =  − .46, 95% CI [− .57, − .35]; low frequency of race communication: AA: *β* =  − .22, 95% CI [− .35, − .07]; CB: *β* =  − .42, 95% CI [− .57, − 26]), and lower bonding was associated with lower grades (high frequency of race communication: AA: *β* = .21, 95% CI [.07, .34]; CB: *β* = .39, 95% CI [.22, .60]; low frequency of race communication: AA: *β* = .24, 95% CI [.09, .39]; CB: *β* = .51, 95% CI [.35, .62]).

#### A-E Discrepancy

For youth with high egalitarianism, for AA youth, higher teacher discrimination was associated with higher discrepancy (*β* = .12, 95% CI [.01, .23]), and higher discrepancy was associated with lower grades (*β* =  − .13, 95% CI [− .25, − .02]), whereas neither path was significant for CB youth. For youth with low egalitarianism, for both AA and CB youth, teacher discrimination was not associated with discrepancy and discrepancy was not associated with grades (*p*s > .05).

For youth AA youth with high preparation for bias, higher teacher discrimination was associated with higher discrepancy (*β* = .11, 95% CI [, .21]), and higher discrepancy was associated with lower grades (*β* =  − .13, 95% CI [− .25, − .02]); for CB youth with high preparation for bias, higher teacher discrimination was associated with higher discrepancy (*β* =  − .16, 95% CI [.01, .35]), but discrepancy was not associated with grades. For youth with low preparation for bias, for both AA and CB youth, teacher discrimination was not associated with discrepancy, and discrepancy was not associated with grades (*p*s > .05).

For youth with high frequency of race communication, for CB youth, higher teacher discrimination was associated with higher discrepancy (*β* = .35, 95% CI [.10, 54]), and higher discrepancy was associated with lower grades (*β* =  − .30, 95% CI [− .48, − .16]); neither path was significant for AA youth. For youth with low frequency of race communication, higher teacher discrimination was not associated with higher discrepancy for AA or CB youth; for AA youth only, higher discrepancy was associated with lower grades (*β* =  − .21, 95% CI [− .34, − .07]).

#### Disciplinary Actions

For youth with high egalitarianism, for AA youth, higher disciplinary actions were associated with lower grades (*β* =  − .21, 95% CI [− .31, − .10]), whereas for CB youth, higher teacher discrimination was associated with higher disciplinary actions (*β* = .38, 95% CI [.23, .50]). For youth with low egalitarianism, for both AA and CB youth, teacher discrimination was not associated with disciplinary actions, and disciplinary actions were not associated with grades (*p*s > .05).

For youth with high preparation for bias, for AA youth, higher teacher discrimination was associated with higher disciplinary actions (*β* = .14,95% CI [.01, .27]), and higher disciplinary actions were associated with lower grades (*β* =  − .22, 95% CI [− .32, − .13]), whereas for CB youth, higher teacher discrimination was associated with higher disciplinary actions (*β* = .35, 95% CI [.23, .50]) but higher disciplinary actions were not associated with grades. For youth with low preparation for bias, for both AA and CB youth, teacher discrimination was not associated with grades directly or through disciplinary actions (*p*s > .05). Of note, for CB youth only, higher disciplinary actions were marginally associated with lower grades (*β* =  − .35, 95% CI [− .64, − .03]).

For youth with high frequency of race communication, for both AA and CB youth, higher disciplinary actions were associated with lower grades (AA: *β* =  − .23, 95% CI [− .36, − .09]; CB: *β* =  − .17, 95% CI [− .32, − .05]). For youth with low frequency of race communication, for both AA and CB youth, higher teacher discrimination was associated with higher disciplinary actions (AA: *β* = .17, 95% CI [.03, .30]; CB: *β* = .39, 95% CI [.07, .60]), and for AA youth only higher disciplinary actions was associated with lower grades (AA: *β* =  − .17, 95% CI [− .27, − .07]).

## Discussion

This study examined the promotive and protective effects of egalitarianism, preparation for bias, and frequency of communication about race for African American and Caribbean Black youth’s school engagement and academic achievement. Both groups reported similar amounts of egalitarianism and preparation for bias messages, as well as similar amounts of communication about race, but ERS messages and communications about race were associated differently with engagement and achievement for these ethnic groups.

### ERS and Communication About Race as Promotive Factors

For African American youth, preparation for bias was associated with better grades but worse school bonding, in line with other research noting that preparation for bias messages may present both benefits and costs (Hughes et al., 2006). One explanation for this seemingly counterintuitive finding is that preparation for bias messages might prompt youth to prepare for and respond to discrimination by working hard in school, whether or not their school bonding is high. This finding highlights the necessity of pairing preparation for bias messages with messages that are less likely to compromise school bonding (e.g., cultural socialization [Wang & Huguley, 2012]). Findings that egalitarianism messages were associated with better grades for African American youth parallels prior research showing that these messages are associated with academic curiosity and persistence (Metzger et al., 2020; Neblett et al., 2006). While preparation for bias messages was associated with less school bonding for Caribbean Black youth, these messages were not connected to their grades, and egalitarianism messages were not associated with engagement or grades for Caribbean Black adolescents. These findings suggest that ERS messages may have different meanings for Black youth, depending on their ethnic background. It has been suggested that ERS effects on school outcomes may be greater for nonimmigrant Black youth for whom there are longer histories of racial bias and oppression within the school context (Wang et al., 2020). Future research with Caribbean Black youth is needed to explore this supposition as most ERS research with Black youth has been conducted with African American samples. Of note, the frequency of conversations about race was associated with better school bonding and less discrepancy between aspirations and expectations for Caribbean Black youth. It is possible that Caribbean Black youth’s conversations about race generally were more salient for their educational experience than preparation for bias and egalitarianism messages. Alternatively, it is possible that simply discussing race, regardless of message content, has benefits for Caribbean Black youth.

### ERS and Communication About Race as Protective Against Teacher Discrimination

Numerous studies have highlighted the toxic effects of teacher discrimination for Black students, including prior research with this sample demonstrating that teacher discrimination is associated with lower school bonding (Kyere et al., 2020), more disciplinary actions (Butler-Barnes & Inniss-Thompson, 2020), and poorer grades (Assari & Caldwell, 2018). Extending that research, we found that more teacher discrimination was associated with a greater discrepancy between aspirations and expectations for African American but not Caribbean Black youth. Relative to Caribbean Black youth, African American youth may have more evidence and awareness that there is a structural ceiling on their educational possibilities given the US opportunity structure. Given aspiration-expectation discrepancies also forecast poorer emotional and behavioral difficulties (e.g., Boxer et al., 2011), efforts to close the A-E discrepancy gap are needed.

Prior research with this sample has shown that the effects of teacher discrimination on grades operate through school bonding (Bryan et al., 2018). We found that increased disciplinary actions and a discrepancy between aspirations and expectations are other links between teacher discrimination and grades. We found minimal evidence that preparation for bias or egalitarianism messages was protective in these pathways. In contrast, when preparation for bias messages and egalitarianism messages was high, the indirect pathway from teacher discrimination to grades through school bonding remained as did the pathway through disciplinary actions for African American youth; these pathways were not evident for low preparation for bias or low egalitarianism messages. It is possible that when egalitarianism messages are met with contradictory treatment from teachers, positive affect toward school diminishes and a connection to school is even more important for students’ grades. Likewise, Saleem et al. ﻿(2022) found a negative association between general discrimination and active coping when egalitarianism was high, and discrimination was associated with stress and lower mastery beliefs in the context of high preparation for bias. Similar findings for the frequency of communication about race emerged for Caribbean Black youth for the teacher discrimination-discrepancy-grades pathway, suggesting that communications about race are not sufficient to protect youth in the face of teacher discrimination. Taken together, the pattern of promotive but not protective effects for egalitarianism and preparation for bias messages, and communication about race, suggests that protection against teacher discrimination requires efforts in addition to traditional ERS.

### Implications

Interventions to promote educational outcomes often focus on improving academic performance (Rathvon, 2008) or enhancing social-emotional skills to support improved academic outcomes (Durlak et al., 2011). Lack of attention to discrimination that Black students encounter at school is a failure to attend to structural factors that impede Black students’ school engagement and achievement, central factors in academic attainment, and multiple adolescent and adult indicators of health and well-being. A decontextualized individual focus may be one factor that explains the limited utility of some interventions implemented with Black youth (Grapin et al., 2019). In addition, simply equipping students with skills places a significant burden on Black youth to adjust in the face of discriminatory practices. Prevention programs must attend to discrimination experiences Black students encounter in the school context. Our findings suggest that these efforts require interventions that directly address teacher discrimination and interventions at the school level to examine and actively work to change bias in systems, policies, and practices (Saleem et al., 2021).

Our findings support a need to integrate ERS messages into interventions to support and enhance the educational experiences of Black youth. Few interventions focus exclusively on ERS (see EMBRace Anderson et al., (2019) as an exception); however, racial socialization has been integrated into family-focused preventive interventions designed to support positive parenting and prevent youth risk behaviors (e.g., Strong African American Families (Murry et al., 2007), Pathways for African American Success (Murry et al., 2018), Black Parenting Strengths and Strategies (Coard et al., 2007). Though school and academic outcomes are not the primary foci of these interventions, the numerous studies showing that positive parenting and reduced risk behaviors forecast better academic outcomes suggest that these approaches may benefit Black youth’s school outcomes. In addition, Stevenson (2014) notes the need for ERS to build racial literacy within the school context in order to combat racial stressors. Universal interventions that attend to cultural and contextual factors (e.g., Ungar et al., 2014), such as ERS, could benefit the entire school racial climate, but in schools with specific concerns or racial disparities, more focused interventions may be needed and at different levels (e.g., students, teachers, administration).

It is important to note that most interventions integrating ERS have focused on “positive” racial socialization (e.g., cultural pride). Less is known about the integration of messages focused on egalitarianism and preparation for bias, the foci of this research. Given youth encounter multiple types of messages about race and ethnicity, interventions designed to promote Black youth’s school success should consider how different combinations of ERS messages impact Black youth’s school outcomes. For example, preparation for bias messages received in the context of cultural socialization and egalitarianism messages has been linked with better academic outcomes for African American youth than preparation for bias received without these other types of messages (Metzger et al., 2020).

While our results highlight important associations between ERS and school engagement and grades, it is important to note that these associations are modest. Given much prior research demonstrating that ethnic-racial socialization benefits Black youth developmental outcomes, we suspect that these associations would be stronger with different measurement strategies (e.g., measures with more items, broader response options, qualitative inquiry). Thus, our work underscores the need for future research to inform practices that use ERS messaging in ways that support Black youth’s school experiences.

### Strengths, Limitations, and Future Directions

A primary strength of this research was examining how ERS is associated with engagement and achievement for African American and Caribbean Black youth. The finding that ERS messages had different associations for African American and Caribbean Black youth underscores the importance of assessing and attending to possible ethnic group differences in the meaning of these messages for Black youth. Simply integrating ERS messages in programs or interventions without attending to heterogeneity within Black youth might yield equivocal effects. This research also extends prior work showing that teacher discrimination adversely affects Black youth’s achievement through school engagement by examining whether ERS messages can disrupt this process.

These study strengths should be considered in the context of some study limitations. Our focus on teacher discrimination neglects other sources of school discrimination such as discrimination from peers and other adults in the school setting (e.g., staff, police) (Montoro et al., 2021). In addition, this research did not assess school policies and procedures that may allow discriminatory practices without censure. In terms of measurement, the items used to assess teacher discrimination did not specify an attribution for the discrimination (e.g., race), though prior research with this sample showed that race was the predominant attribution for Black youth’s perception of discriminatory experiences (Seaton et al., 2010). Our measure of preparation for bias and egalitarianism messages only assessed whether or not these messages were received, not how much of these messages were received, or whether the messages were oriented toward discrimination experiences that could occur at school. Scott and colleagues (2020) note the problems with decontextualizing preparation for bias messages noting that these messages might be tailored to specific situations, and that the consequences of preparation for bias messages may differ depending on the context. Another measurement concern is that some constructs included only a small number of items, possibly obscuring important findings. Also, our measure of disciplinary actions included time at a reform school, detention center, jail, or prison; though these often are an escalation of disciplinary actions from schools, the result of harsh discipline policies, or actions imposed as alternatives to school, we acknowledge that these are not necessarily direct measures of school disciplinary action. Finally, ﻿the primary study constructs were assessed via youth self-report, possibly yielding a self-report bias in our results. While we are unaware of other ways to assess youth’s experience of teacher discrimination, multiple reporters of study constructs and use of varied methodologies to assess these constructs is an important need for future research.

A notable strength of this research was considering ethnic heterogeneity among Black youth. However, we were unable to explore differences within Caribbean Black youth due to sample size constraints. Approximately 60% of the Caribbean Black youth in this sample were born in the USA. For youth born outside the USA, country of origin and length of time in the USA could influence the type of ERS messages that are important. Similarly, parent nativity is important to consider in understanding heterogeneity within Caribbean Black youth. In addition to ethnic differences, the school engagement, achievement, and attainment of male and female Black youth vary (e.g., de Brey et al., 2019). Future research that attends to the intersection of ethnicity and sex within Black youth is needed to advance our understanding of the effects of teacher discrimination on Black youth, and to inform the design of preventive interventions that reflect the diversity within Black youth.

Given the cross-sectional design, we were unable to infer causality. Numerous longitudinal studies have identified discrimination as a predictor of Black youth’s academic, social, and behavioral outcomes, increasing our confidence that teacher discrimination initiates a process that can derail Black youth’s school outcomes. Nonetheless, longitudinal data are needed to clarify these associations. In addition, we acknowledge that our strategy for exploring moderated mediation was dependent on the measurement structure (e.g., binary); future research should test these pathways with more stringent tests. Finally, it is important to note that these data were collected between 2001 and 2003. Contemporary youth live in a different political, social, and environmental landscape, with increased media presence and influence. Future research should include contemporaneous samples of Black youth, including those of Caribbean descent, so that we are able to have a more contextually relevant understanding of these youth’s discrimination experiences and the effects. Limitations notwithstanding, these data which include both African American and Caribbean Black youth, present an unparalleled opportunity to explore discrimination, ERS, and educational experiences among Black youth. To date, there are no other data sources that afford researchers the opportunity to examine these issues among Caribbean Black youth. Given the persistence of discrimination, study findings remain relevant to prevention and intervention planning to promote better educational experiences among today’s generation of Black youth.

## Supplementary Information

Below is the link to the electronic supplementary material.

Supplementary file1 (DOCX 172 KB)

## Data Availability

These data are publicly available at https://www.icpsr.umich.edu/web/RCMD/studies/36380.

## Footnotes

1 The same pattern of results was obtained when examining the three mediators in the same model.
