# Supplementary material for: Ethnic-Racial Socialization, Teacher Discrimination, and Black Youth’s School Engagement and Achievement
Source: Prev Sci. 2023 Jun 7;25(1):56–67. doi: 10.1007/s11121-023-01551-z (PMC11126456; doi:10.1007/s11121-023-01551-z)
Supplement: Supplementary file 1 — Supplementary file1 (DOCX 172 KB) [file 11121_2023_1551_MOESM1_ESM.docx]

Table 1

*Unweighted and Weighted Demographic Characteristics of the Sample by Ethnicity*

| Demographic Characteristic | African American  n^a^ = 810 | | | Caribbean Black  n^a^ = 360 | | | Characteristic x Ethnicity |
| --- | --- | --- | --- | --- | --- | --- | --- |
|  | n^a^ | %uwt^b^ | %wt(SE)^c^ | n^a^ | %uwt^b^ | %wt(SE)^c^ | χ^2^ |
| Gender |  |  |  |  |  |  |  |
| Male | 398 | 49.1 | 50.4(1.9) | 165 | 45.8 | 44.8(2.3) | χ^2^ = 3.48  *p* = .07 |
| Female | 412 | 50.9 | 49.6(1.9) | 195 | 54.2 | 55.2(2.3) |  |
| Age |  |  |  |  |  |  |  |
| 13 | 165 | 20.4 | 20.8(1.9) | 56 | 15.6 | 10.6(1.2) | χ^2^ = 3.51  *p* = .04 |
| 14 | 184 | 22.7 | 20.1(1.6) | 72 | 20.0 | 20.2(1.4) |  |
| 15 | 135 | 16.7 | 20.0(1.6) | 79 | 21.9 | 28.3(4.3) |  |
| 16 | 153 | 18.9 | 20.2(1.8) | 74 | 20.6 | 18.4(4.4) |  |
| 17 | 173 | 21.4 | 18.9(1.2) | 79 | 21.9 | 22.5(1.1) |  |
| Household Income^d^ |  |  |  |  |  |  |  |
| $0 - $17,999 | 244 | 30.2 | 27.7(2.5) | 67 | 18.8 | 26.2(5.2) | χ^2^ = 0.97  *p* = .36 |
| $18,000 - $31,999 | 223 | 27.6 | 27.4(1.8) | 107 | 30.0 | 20.3(3.2) |  |
| $32,000 - $54,999 | 187 | 23.2 | 24.6(2.4) | 94 | 26.3 | 33.2(4.2) |  |
| ≥ $55,000 | 153 | 19.0 | 20.2(2.2) | 89 | 24.9 | 20.3(6.7) |  |
| Region^d^ |  |  |  |  |  |  |  |
| Northeast | 87 | 10.8 | 13.4(1.6) | 251 | 70.3 | 63.9(2.7) | χ^2^ = 48.90  *p* < .001 |
| Midwβ | 101 | 12.5 | 15.7(2.2) | 2 | 0.6 | 3.7(0.7) |  |
| South | 569 | 70.3 | 61.8(2.7) | 104 | 29.1 | 32.4(2.1) |  |
| Wβ | 52 | 6.4 | 9.1(1.8) | 0 | 0 | 0(0) |  |
| Parent Nativity^d^ |  |  |  |  |  |  |  |
| Born in the U.S. | 788 | 98.0 | 97.7(0.6) | 66 | 20.2 | 20.4(3.2) | χ^2^ = 722.81  *p* < .001 |
| Born outside the U.S. | 16 | 2.0 | 2.3(0.6) | 260 | 79.8 | 79.6(3.2) |  |
| Parent Work Status^d^ |  |  |  |  |  |  |  |
| Not Working | 215 | 26.6 | 26.2(2.7) | 69 | 19.3 | 24.5(12.6) | χ^2^ = 0.02  *p =* .89 |
| Working | 593 | 73.4 | 73.8(2.7) | 288 | 80.7 | 75.5(12.6) |  |

*Note.* All weighted βimates are weighted to be nationally representative of the given population and subpopulations in the contiguous 48 states of the United States. Standard errors and χ^2^ statistics are adjusted for the sampling stratification, clustering, and weighting of the data.

^a^Sample size (n) is unweighted

^b^Unweighted

^c^Weighted (standard error)

^d^Reflects adult respondents’ status

Table 2

*Partial Correlations among Study Variables after Controlling for Covariates (Age, Sex, Family Income, and Parental Education), by Ethnicity*

|  | 1 | 2 | 3 | 4 | 5 | 6 | 7 | 8 |
| --- | --- | --- | --- | --- | --- | --- | --- | --- |
| 1. Egalitarianism | -- | .07^*^ | -.01 | -.02 | .07^+^ | -.01 | -.11^**^ | .12^**^ |
| 1. Preparation for Bias | -.04 | -- | .10^*^ | .21^**^ | -.11^**^ | .05 | .02 | .08^*^ |
| 1. Race Communication | 0 | .16^**^ | -- | .05 | .08^*^ | -.03 | -.03 | .01 |
| 1. Teacher Discrimination | -.06 | .18^**^ | -.01 | -- | -.25^**^ | .11^**^ | .16^**^ | -.06^+^ |
| 1. School Bonding | -.04 | -.20^**^ | .14^**^ | -.30^**^ | -- | -.08^*^ | -.23^**^ | .25^**^ |
| 1. Discrepancy | -.03 | .12^*^ | -.14^*^ | .04 | -.17^**^ | -- | .08^*^ | -.11^**^ |
| 1. Disciplinary Action | -.03 | .19^**^ | .05 | .17^**^ | -.21^**^ | .08 | -- | -.20^**^ |
| 1. Grades | .04 | -.09 | .11^*^ | -.07 | .45^**^ | -.19^**^ | -.21^**^ | -- |

*Note.* Correlations for African American above diagonal; correlations for Caribbean Black below diagonal.

Race Communication = Frequency of Race Communication.

^+^ *p* < .10. ^*^ *p* < .05. ^**^ *p* < .01.

Table 3

*Results from the Multigroup Analyses by Ethnicity for School Bonding as a Mediator*

|  | African American | | | | |  | Caribbean Black | | | | |
| --- | --- | --- | --- | --- | --- | --- | --- | --- | --- | --- | --- |
| Moderator: Egalitarianism | Low Egal | |  | High Egal | |  | Low Egal | |  | High Egal | |
|  | β | 95% CI |  | β | 95% CI |  | β | 95% CI |  | β | 95% CI |
| Teacher discrimination 🡪 Mediator | **-.38** | **-.51 – -.26** |  | **-.19** | **-.28 – -.09** |  | **-.45** | **-.59 – -.21** |  | **-.45** | **-.57 – -.34** |
| Mediator 🡪 Grades | .17 | -.05 – .37 |  | **.24** | **.14 – .33** |  | .29 | -.01 – .55 |  | **.55** | **.43 – .69** |
| Indirect Effect | -.07 | -.13 – .02 |  | **-.05** | **-.07 – -.02** |  | -.13 | -.29 – .01 |  | **-.25** | **-.37 – -.17** |
| Moderator: Preparation for Bias | Low Prep Bias | |  | High Prep Bias | |  | Low Prep Bias | |  | High Prep Bias | |
|  | β | 95% CI |  | β | 95% CI |  | β | 95% CI |  | β | 95% CI |
| Teacher discrimination 🡪 Mediator | **-.22** | **-.38 – -.05** |  | **-.23** | **-.26 – -.10** |  | **-.44** | **-.63 – -.11** |  | **-.44** | **-.53 – -.27** |
| Mediator 🡪 Grades | **.25** | **.06 – .39** |  | **.23** | **.13 – .33** |  | **.61** | **.12 – .82** |  | **.46** | **.38 – .53** |
| Indirect Effect | **-.05** | **-.12 – -.004** |  | **-.05** | **-.07 – -.02** |  | **-.27** | **-.48 – -.02** |  | **-.20** | **-.26 – -.11** |
| Moderator: Race Communication | Low Race Comm | |  | High Race Comm | |  | Low Race Comm | |  | High Race Comm | |
|  | β | 95% CI |  | β | 95% CI |  | β | 95% CI |  | β | 95% CI |
| Teacher discrimination 🡪 Mediator | **-.22** | **-.35 – -.07** |  | **-.27** | **-.32 – -.20** |  | **-.42** | **-.57 – -.26** |  | **-.46** | **-.57 – -.35** |
| Mediator 🡪 Grades | **.24** | **.09 – .39** |  | **.21** | **.07 – .34** |  | **.51** | **.35 – .62** |  | **.39** | **.22 – .60** |
| Indirect Effect | **-.05** | **-.11 – -.01** |  | **-.06** | **-.09 – -.02** |  | **-.21** | **-.31 – -.12** |  | **-.18** | **-.28 – -.11** |

*Note.* Bold indicates path is significant.

Table 4

*Results from the Multigroup Analyses by Ethnicity for Aspiration-Expectation Discrepancy*

|  | African American | | | | |  | Caribbean Black | | | | |
| --- | --- | --- | --- | --- | --- | --- | --- | --- | --- | --- | --- |
| Moderator: Egalitarianism | Low Egal | |  | High Egal | |  | Low Egal | |  | High Egal | |
|  | β | 95% CI |  | β | 95% CI |  | β | 95% CI |  | β | 95% CI |
| Teacher discrimination 🡪 Mediator | .10 | -.22 – .32 |  | **.12** | **.01 – .23** |  | .21 | -.14 – .54 |  | .10 | -.02 – .26 |
| Mediator 🡪 Grades | -.07 | -.29 – .17 |  | **-.13** | **-.25 – -.01** |  | -.33 | -.70 – .01 |  | -.12 | -.34 – .11 |
| Indirect Effect | -.01 | -.06 – .03 |  | **-.02** | **-.05 – 0** |  | -.07 | -.35 – .02 |  | -.01 | -.06 – .02 |
| Moderator: Preparation for Bias | Low Prep Bias | |  | High Prep Bias | |  | Low Prep Bias | |  | High Prep Bias | |
|  | β | 95% CI |  | β | 95% CI |  | β | 95% CI |  | β | 95% CI |
| Teacher discrimination 🡪 Mediator | .13 | -.27 – .46 |  | **.11** | **0 – .21** |  | -.31 | -.98 – .23 |  | **.16** | **.01 – .35** |
| Mediator 🡪 Grades | -.05 | -.36 – .24 |  | **-.13** | **-.25 – -.02** |  | -.31 | -2.99 – .78 |  | -.14 | -.31 – .02 |
| Indirect Effect | -.01 | -.11 – .05 |  | **-.01** | **-.04 – 0** |  | .10 | -.77 – 2.86 |  | -.02 | -.05 – .01 |
| Moderator: Race Communication | Low Race Comm | |  | High Race Comm | |  | Low Race Comm | |  | High Race Comm | |
|  | β | 95% CI |  | β | 95% CI |  | β | 95% CI |  | β | 95% CI |
| Teacher discrimination 🡪 Mediator | .12 | -.06 – .26 |  | .12 | -.02 – .25 |  | -.12 | -.40 – .16 |  | **.35** | **.10 – .54** |
| Mediator 🡪 Grades | **-.21** | **-.34 – -.07** |  | -.07 | -.22 – .07 |  | -.09 | -.32 – .04 |  | **-.30** | **-.48 – -.16** |
| Indirect Effect | -.03 | -.07 – .01 |  | -.01 | -.04 – .01 |  | .01 | -.03 – .05 |  | **-.11** | **-.21 – -.02** |

*Note.* Bold indicates path is significant.

Table 5

*Results from the Multigroup Analyses by Ethnicity for Mediator Disciplinary Action*

|  | African American | | | | |  | Caribbean Black | | | | |
| --- | --- | --- | --- | --- | --- | --- | --- | --- | --- | --- | --- |
| Moderator: Egalitarianism | Low Egal | |  | High Egal | |  | Low Egal | |  | High Egal | |
|  | β | 95% CI |  | β | 95% CI |  | β | 95% CI |  | β | 95% CI |
| Teacher discrimination 🡪 Mediator | .18 | -.03 – .39 |  | .13 | -.02 – .26 |  | .09 | -.23 – .58 |  | **.38** | **.23 – .50** |
| Mediator 🡪 Grades | -.14 | -.28 – .04 |  | **-.21** | **-.31 – -.10** |  | -.41 | -.69 – .02 |  | -.09 | -.20 – .01 |
| Indirect Effect | -.02 | -.07 – .01 |  | -.03 | -.07 – .003 |  | -.04 | -.29 – .06 |  | -.03 | -.08 – 0 |
| Moderator: Preparation for Bias | Low Prep Bias | |  | High Prep Bias | |  | Low Prep Bias | |  | High Prep Bias | |
|  | β | 95% CI |  | β | 95% CI |  | β | 95% CI |  | β | 95% CI |
| Teacher discrimination 🡪 Mediator | .12 | -.07 – .34 |  | .14 | .01 – .27 |  | -.02 | -.42 – .41 |  | .35 | .23 – .50 |
| Mediator 🡪 Grades | -.09 | -.33 – .18 |  | -.22 | -.32 – -.13 |  | -.35 | -.64 – -.03 |  | -.12 | -.31 – .03 |
| Indirect Effect | -.01 | -.07 – .02 |  | -.03 | -.07 – -.002 |  | .01 | -.21 – .15 |  | -.04 | -.14 – .01 |
| Moderator: Race Communication | Low Race Comm | |  | High Race Comm | |  | Low Race Comm | |  | High Race Comm | |
|  | β | 95% CI |  | β | 95% CI |  | β | 95% CI |  | β | 95% CI |
| Teacher discrimination 🡪 Mediator | **.17** | **.03 – .30** |  | .13 | -.03 – .28 |  | **.39** | **.07 – .60** |  | .23 | -.21 – .51 |
| Mediator 🡪 Grades | **-.17** | **-.27 – -.07** |  | **-.23** | **-.36 – -.09** |  | -.19 | -.46 – .08 |  | **-.17** | **-.32 – -.05** |
| Indirect Effect | -.03 | -.06 – -.004 |  | -.03 | -.08 – .01 |  | -.07 | -.25 – .01 |  | -.04 | -.14 – .04 |

*Note.* Bold indicates path is significant.


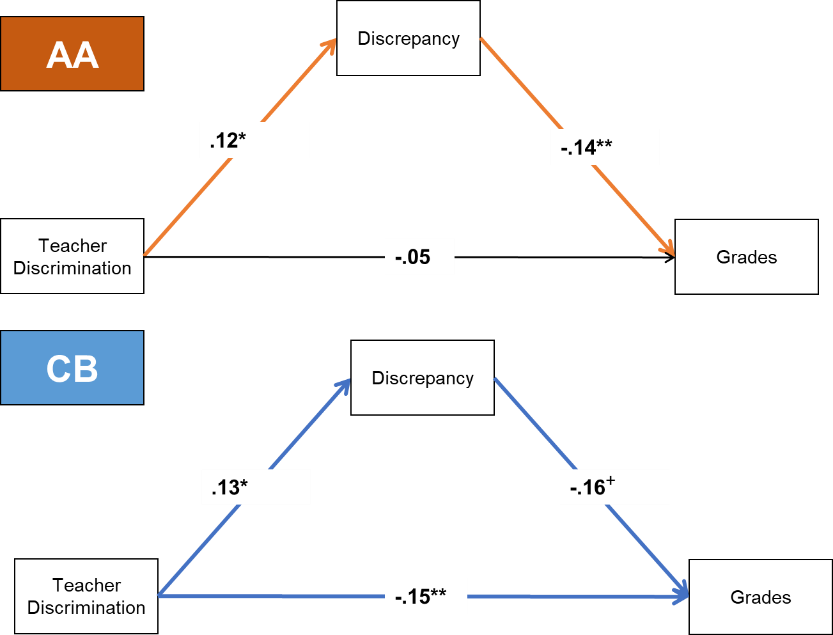

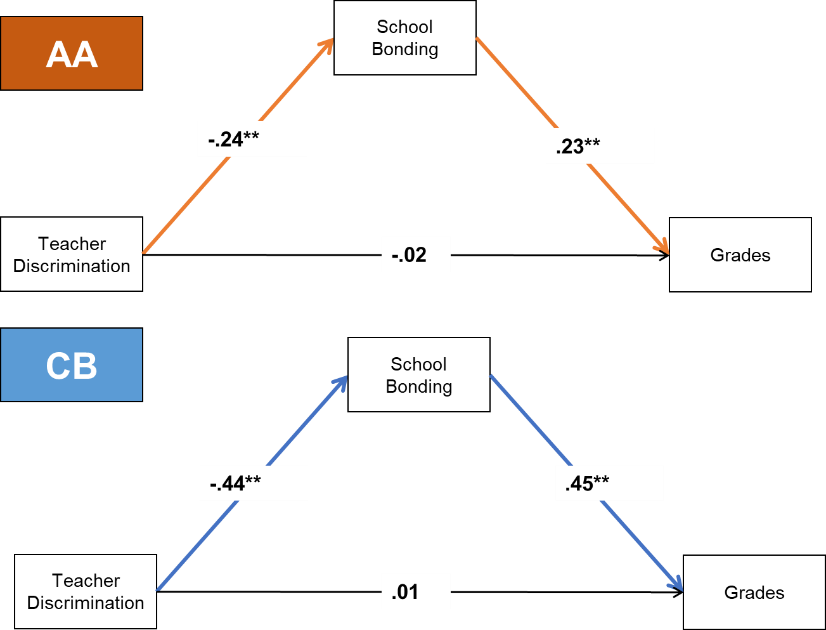


Figure 1c


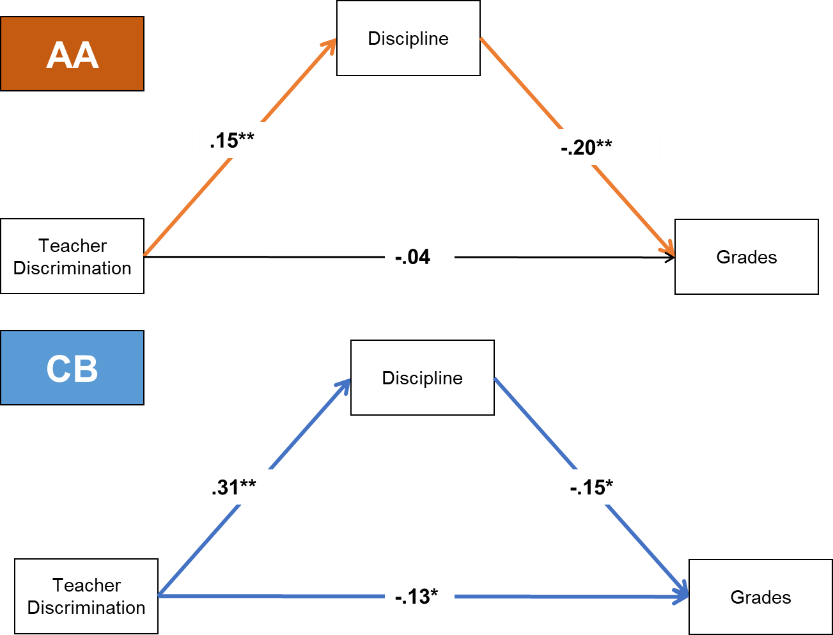


*Figure 1.* Structural equation model estimating the effect of teacher discrimination on adolescents’ grades mediated by adolescents’ school engagement
